# Pharmacological increases in circulating ketones fail to alleviate the hypertrophic cardiomyopathy present in the Tafazzin knockdown mouse model of Barth syndrome

**DOI:** 10.3389/jpps.2025.15688

**Published:** 2025-12-04

**Authors:** Tanin Shafaati, Amanda A. Greenwell, Christina T. Saed, Seyed Amirhossein Tabatabaei Dakhili, Jordan S. F. Chan, Linyue Dong, Magnus J. Stenlund, Sally R. Ferrari, Ruth Han, Jennifer Kruger, Farah Eaton, Keshav Gopal, Sandra T. Davidge, Gavin Y. Oudit, John R. Ussher

**Affiliations:** 1 Faculty of Pharmacy and Pharmaceutical Sciences, University of Alberta, Edmonton, AB, Canada; 2 Cardiovascular Research Centre, University of Alberta, Edmonton, AB, Canada; 3 Women and Children’s Health Research Institute, University of Alberta, Edmonton, AB, Canada; 4 Health Sciences Laboratory Animal Services, University of Alberta, Edmonton, AB, Canada; 5 Department of Obstetrics and Gynaecology, Faculty of Medicine and Dentistry, University of Alberta, Edmonton, AB, Canada; 6 Divsion of Cardiology, Department of Medicine, Faculty of Medicine and Dentistry, University of Alberta, Edmonton, AB, Canada; 7 Mazankowski Alberta Heart Institute, University of Alberta, Edmonton, AB, Canada

**Keywords:** Barth syndrome, cardiomyopathy, ketones, empagliflozin, substrate metabolism

## Abstract

**Objective:**

Mutations in the *tafazzin* gene lead to impaired remodeling of cardiolipin, thereby impairing mitochondrial function and causing Barth syndrome (BTHS), a rare X-linked genetic disorder characterized by cardiomyopathy. Previous studies in a mouse model of BTHS, secondary to knockdown of *Tafazzin* (TazKD mice), also observed perturbations in mitochondrial substrate metabolism and a hypertrophic cardiomyopathy. BTHS may be characterized by increased cardiac ketone metabolism, as myocardial protein expression of the ketolytic enzyme, β-hydroxybutyrate dehydrogenase 1 (BDH1), was markedly increased in TazKD mice. We therefore determined whether increasing ketone supply in TazKD mice may have therapeutic utility against their cardiac abnormalities.

**Methods:**

We treated TazKD mice and their wild-type littermates with either the sodium-glucose cotransporter-2 inhibitor, empagliflozin (10 mg/kg), or a ketone ester (KE; 1719 mg/kg) once daily for 7-week, and performed ultrasound echocardiography to assess cardiac structure and function.

**Results:**

Treatment of TazKD mice with either empagliflozin or a KE increased circulating ketone levels. However, neither approach proved capable of alleviating the cardiac hypertrophy present in TazKD mice, as their increased left ventricular wall thickness and decreased left ventricular diameter remained comparable to that observed in vehicle control treated animals. We also observed that empagliflozin and KE treatment did not impact key markers of cardiac hypertrophy in TazKD mice.

**Conclusion:**

Increasing circulating ketone levels did not alleviate the cardiac hypertrophy in TazKD mice, suggesting that such an approach would not improve outcomes in BTHS.

## Introduction

Barth Syndrome (BTHS) is a rare genetic disease due to mutations in *tafazzin*, a gene on chromosome Xq28.12 that encodes for the protein tafazzin, which plays a key role in the remodeling of cardiolipin (CL). Infantile-onset cardiomyopathy often resulting in heart failure is the dominant clinical manifestation in people living with BTHS, though other adverse effects including neutropenia, exercise intolerance, and 3-methylglutaconic aciduria are also often present [1, 2]. Because proper remodeling of CL is critical to optimal electron transport chain (ETC) function, BTHS is also characterized by several perturbations in energy metabolism and underlying mitochondrial dysfunction.

At the level of the heart, this includes a reduction in glucose oxidation in mice mimicking BTHS due to whole-body knockdown of *Tafazzin* (herein referred to as TazKD mice) [3]. Furthermore, several studies have reported reductions in myocardial fatty acid oxidation in TazKD mice [4, 5]. Metabolomic profiling also suggests perturbations in ketone metabolism in people living with BTHS [6], though we did not observe changes in myocardial ketone [β-hydroxybutyrate (βOHB)] oxidation rates in TazKD mice [3]. However, the similar myocardial ketone oxidation rates in *ex vivo* isolated working heart perfusions from TazKD mice were at fixed βOHB concentrations when compared to their wild-type (WT) littermates, whereas myocardial protein expression of the ketolytic enzyme, βOHB dehydrogenase 1 (BDH1), was markedly increased in TazKD mice. These findings are consistent with the elevated myocardial BDH1 expression and increased ketone oxidation observed in animals and humans with heart failure [7, 8]. Importantly, circulating ketone supply is a key determinant of myocardial ketone oxidation rates [9]. Hence, we reasoned that although isolated working hearts from TazKD mice exhibit similar ketone oxidation rates at fixed βOHB concentrations, their capacity to oxidize ketones would be greater in response to therapeutic and/or nutritional approaches that increase circulating ketones.

The sodium-glucose cotransporter-2 (SGLT2) inhibitors are a newer glucose-lowering drug class used to treat type 2 diabetes that have been shown to improve cardiovascular outcomes [10]. As SGLT2 inhibitors are known to frequently increase circulating ketones in both animals and humans, increases in myocardial ketone metabolism may explain some of their cardioprotective actions, though this is an ongoing topic of debate [11]. Another strategy that is often pursued to increase circulating ketones involves adherence to a ketogenic dietary pattern [12]. Since ketogenic diets present other issues such as increased risk of dyslipidemia and long-term adherence concerns, other dietary strategies to increase circulating ketones levels have been explored, with drinkable ketone esters (KEs) for oral consumption emerging as a popular approach [13–15]. Given the observed increase in myocardial BDH1 protein expression in TazKD mice and conjecture that increased ketone metabolism may benefit the failing heart, we hypothesized that augmenting ketone metabolism may have utility in alleviating BTHS-related cardiomyopathy. Accordingly, we investigated whether increasing circulating ketone levels via either administration of empagliflozin or KEs would impact cardiovascular parameters in TazKD mice.

## Methods

### Animal care and experimentation

**FIGURE 1:**
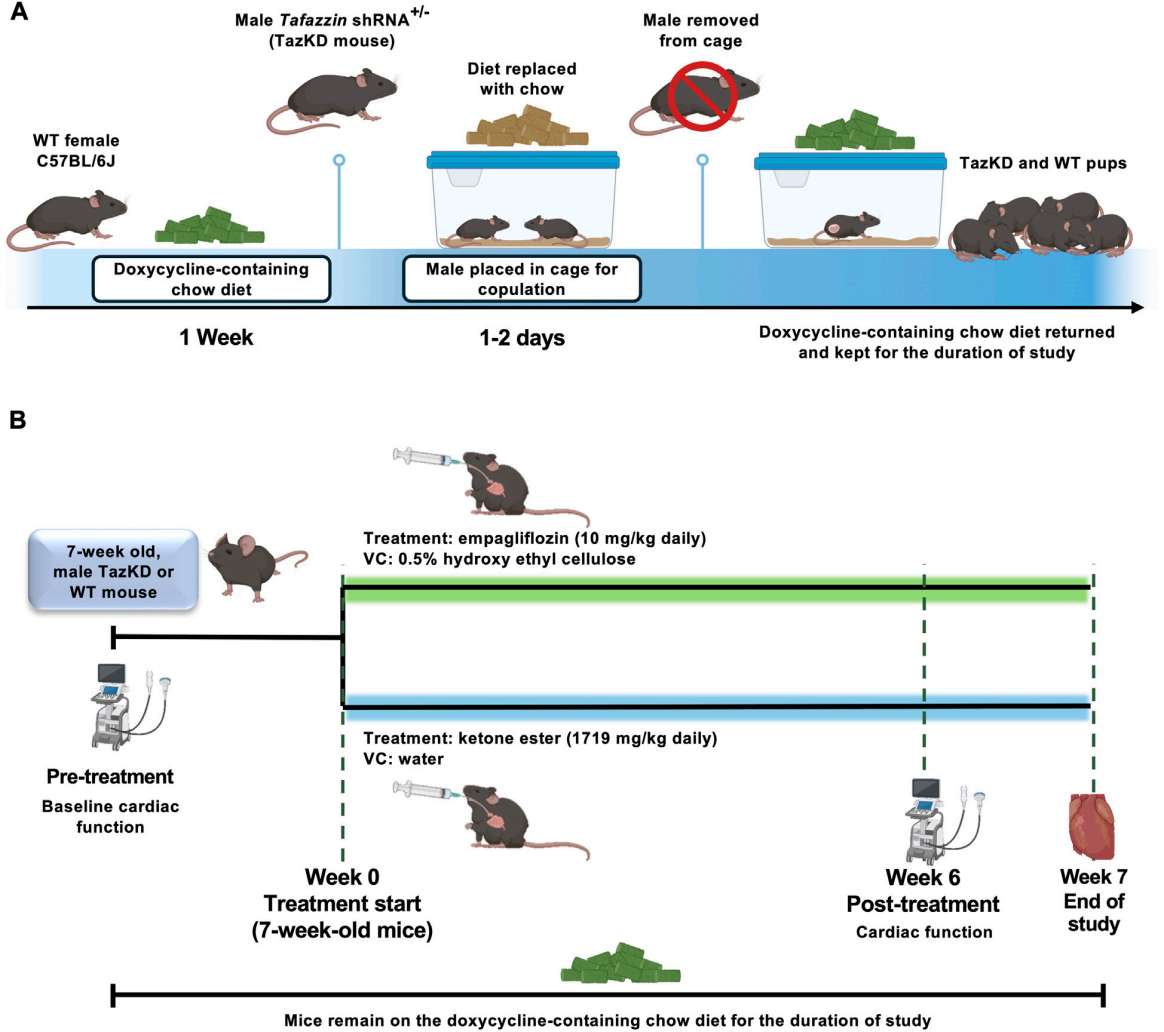
**(A)** Breeding strategy for doxycycline-inducible TazKD mice. Female C57BL/6J mice were maintained on a doxycycline-containing diet for 1-week prior to breeding with males heterozygous for the transgene encoding for the *Tafazzin* shRNA. During mating, doxycycline chow was temporarily replaced with standard chow to avoid effects on male fertility. Following confirmation of copulatory plugs, males were removed, and doxycycline chow was reinstated to maintain *Tafazzin* knockdown during embryogenesis and throughout the study. **(B)** Experimental interventions and study design. Ultrasound echocardiography was used to assess baseline cardiac function of TazKD mice and WT littermates, following which animals were split into experimental arms for treatment with empagliflozin (10 mg/kg) or KE (1719 mg/kg) daily via oral gavage for 7-week. Cardiac function was reassessed following 6-week of treatment, with animals being euthanized 1-week after the final assessment of cardiac function. shRNA, short-hairpin RNA; VC, vehicle control; WT, Wild-type.

All procedures were approved by the University of Alberta Health Sciences Animal Welfare Committee and abided to the Canadian Council on Animal Care guidelines. Mice were housed at 22 °C with a 12-h light/dark cycle, standard enrichment, and had *ad libitum* access to food and water. The doxycycline-inducible TazKD mouse model was generated as described [16], with all mice receiving a doxycycline-containing (625 mg/kg) chow diet throughout the study to induce sufficient short-hairpin RNA mediated knockdown. Female C57BL/6J mice were placed on doxycycline diet 1 week prior to breeding with males heterozygous for the transgene encoding for the *Tafazzin* short-hairpin RNA. Once the male was added to the cage to initiate copulation with the female, the doxycycline diet was replaced with standard chow as doxycycline has been reported to interfere with male fertility [17]. Once copulatory plugs were detected in the females (usually within 1–2 days of mating), the male breeders were removed from the cages and the doxycycline diet was returned (Figure 1). The doxycycline diet was provided 1 week prior to mating to ensure that circulating doxycycline concentrations were at a sufficient level to induce *Tafazzin* knockdown upon conception and embryogenesis. PCR genotyping to confirm the presence or absence of the transgene encoding for the *Tafazzin* short-hairpin RNA was performed in toe clip biopsies from all offspring at ∼7 days as we have previously described [18]. Male littermates lacking the transgene were maintained on doxycycline chow and served as the WT littermate controls. 7-week-old male TazKD and WT mice were randomized to 7-week of treatment with empagliflozin (MedChemExpress, 10 mg/kg, dissolved in 0.5% hydroxyethyl cellulose) or vehicle control (VC; 0.5% hydroxyethyl cellulose) once daily via oral gavage. Another group of 7-week-old male TazKD and WT mice were randomized to once daily treatment for 7-week with a commercially available KE supplement drink ((R)-3-hydroxybutyl (R)-3-hydroxybutyrate; deltaG Ketones, 1719 mg/kg, dissolved in sterilized Milli-Q water) or VC (water) via oral gavage. The KE was dissolved at a concentration of 533 mg/mL in sterilized Milli-Q water, with the volume gavaged once daily in mice ranging anywhere from ∼80 to 150 μL. To verify whether our treatments were increasing ketones, circulating βOHB levels were measured following 3-week of treatment from tail whole blood using the FreeStyle Precision Neo blood ketone monitoring system (Abbott). At study completion, all mice were euthanized by an intraperitoneal injection of sodium pentobarbital (12 mg), following which peripheral tissues were rapidly excised and snap-frozen using liquid nitrogen-cooled Wollenberger tongs and stored at −80 °C.

### Ultrasound echocardiography

Cardiac ultrasound images were acquired using a VisualSonics Vevo 3100 and MX 550S probe as previously described [19, 20]. Mice were anesthetized with 2–3% isoflurane initially and then maintained at 1–1.5% during imaging, with body temperature, respiration, and heart rate monitored throughout the procedure. Cardiac structure and left ventricular (LV) function were assessed in 7-week-old WT and TazKD mice at baseline and post 7-week empagliflozin or KE treatment. Parameters assessed included LV diameter and volume, anterior and posterior wall thickness, ejection fraction, fractional shortening and cardiac output.

### Real-time quantitative PCR (qPCR)

First-strand complementary DNA (cDNA) was generated from 2 µg of RNA isolated from ∼15 mg of powdered frozen heart tissue using the High-Capacity cDNA Reverse Transcription Kit (Applied Biosystems, 4368814), following the manufacturer’s protocol. qPCR was performed on a Bio-Rad CFX Connect using SYBR Green (64625020, Bio-Rad). 18S ribosomal RNA (18S rRNA) was used as the internal housekeeping gene, with relative mRNA expression levels determined using the 2^−ΔΔCT^ method as previously described [21]. Primer sequences for measured genes are provided below.

*Rna18s* forward: TAG AGG GAC AAG TGG CGT TC
*Rna18s* reverse: CGC TGA GCC AGT CAG TGT
*Myh7* forward: CCG AGT CCC AGG TCA ACA A
*Myh7* reverse: CTT CAC GGG CAC CCT TGG A
*Acta1* forward: CGA CGG GCA GGT CAT CA
*Acta1* reverse: ACC GAT AAA GGA AGG CTG GAA
*Nppb* forward: GAG GTC ACT CCT ATC CTC TGG
*Nppb* reverse: GCC ATT TCC TCC GAC TTT TCT C
*Nppa* forward: ACC TGC TAG ACC ACC TGG AG
*Nppa* reverse: CCT TGG CTG TTA TCT TCG GTA CCG G

### Statistical analysis

Data are presented as mean ± SEM. Statistical significance was determined by two-way ANOVA, with differences considered significant when *P* < 0.05. Fisher’s Least Significant Difference (LSD) was selected as the post-hoc analysis. Analyses were performed using GraphPad Prism 10 software.

## Results

### Treatment with empagliflozin increases circulating βOHB levels in TazKD mice but does not improve parameters of cardiac function and structure

**FIGURE 2:**
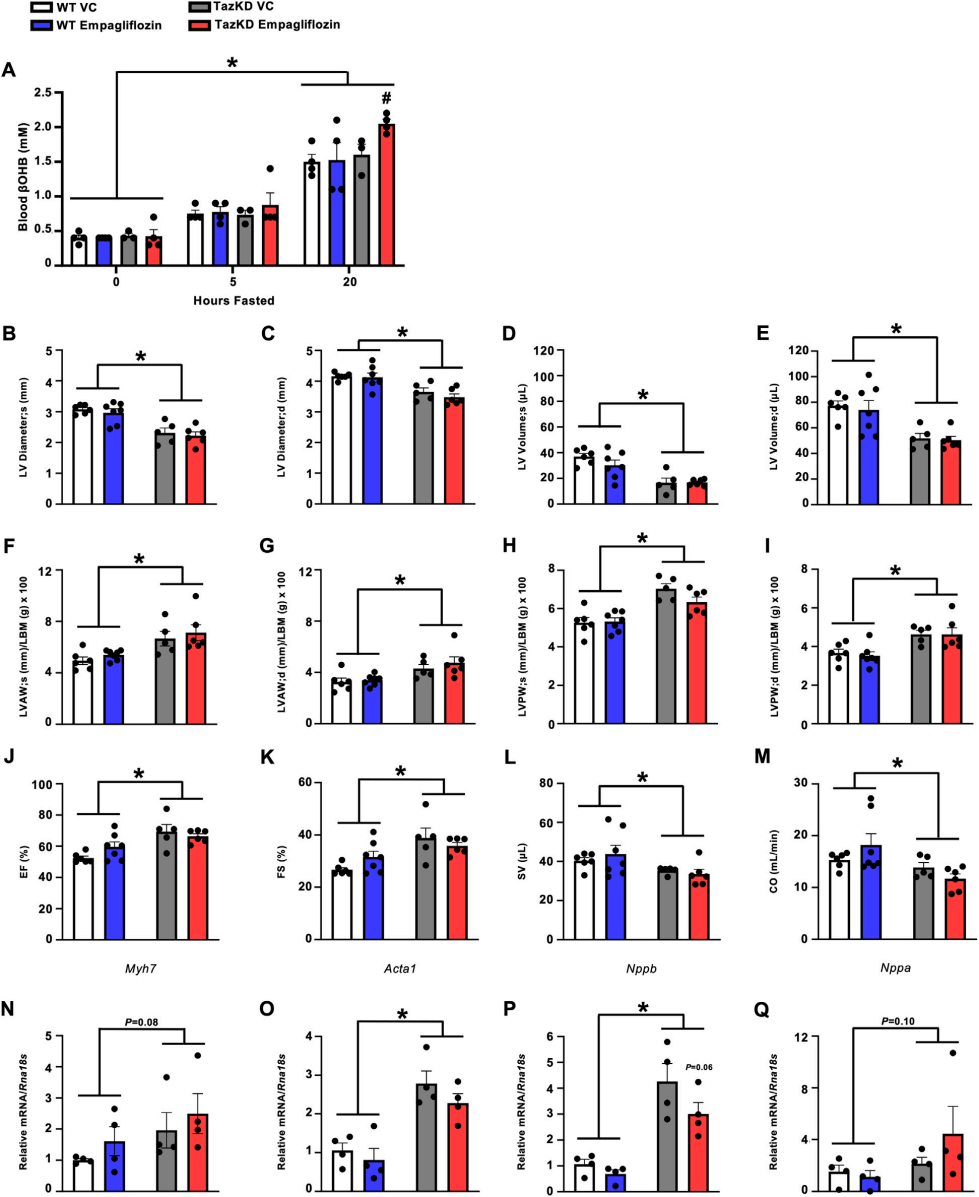
Empagliflozin treatment increases circulating βOHB levels in fasted TazKD mice but does not alleviate the cardiac abnormalities in TazKD mice. **(A)** Circulating βOHB levels measured at several timepoints during fasting (n = 4). Ultrasound echocardiography was used to evaluate **(B,C)** LV diameter during systole and diastole, **(D,E)** LV volume during systole and diastole, **(F,G)** LVAW thickness during systole and diastole, **(H,I)** LVPW thickness during systole and diastole, **(J)** LVEF, **(K)** LVFS, **(L)** SV, and **(M)** CO in TazKD and WT mice treated with VC or empagliflozin (n = 5–7). Myocardial mRNA expression of **(N)** *Myh7*, **(O)** *Acta1*, **(P)** *Nppb*, and **(Q)** *Nppa* relative to *Rna18s* (n = 4). All values are presented as mean ± SEM. *P < 0.05, significantly different versus WT mice. ^#^ *P* < 0.05, significantly different versus VC treated counterpart. ^$^ *P* < 0.05, significantly different versus other timepoints. *Acta1*, skeletal muscle α-actin; βOHB, β-hydroxybutyrate; CO, cardiac output; d, diastole; KE, ketone ester; LBM, lean body mass; LV, left ventricular; LVAW, left ventricular anterior wall; LVEF, left ventricular ejection fraction; LVFS, left ventricular fractional shortening; LVPW, left ventricular posterior wall; *Myh7*, myosin heavy chain 7; *Nppa*, atrial natriuretic peptide; *Nppb*, brain natriuretic peptide; *Rna18S*, 18S ribosomal RNA; s, systole; SV, stroke volume; VC, vehicle control; WT, Wild-type.

7-week-old TazKD mice and their WT littermates were treated once daily via oral gavage for 7-week with the SGLT2 inhibitor, empagliflozin (10 mg/kg), or VC. To confirm that empagliflozin was increasing circulating ketones, blood βOHB levels were measured following 3-week of treatment in all mice during the random fed state, and after 5 or 20 h of fasting. While treatment with empagliflozin did not increase circulating βOHB levels in WT mice, it did increase circulating βOHB levels in fasted TazKD mice (Figure 2A).

In opposition of our hypothesis, treatment with empagliflozin did not improve the structural perturbations present in the hearts of TazKD mice. TazKD mice presented with a smaller LV diameter which was associated with lower LV volumes, though treatment with empagliflozin did not improve any of these parameters (Figures 2B–E). Furthermore, LV anterior and posterior walls were thickened in TazKD mice but once again unaltered by empagliflozin treatment (Figures 2F–I). Consistent with our previous observations and that of others [20], TazKD mice also displayed a mild increase in LVEF and LVFS, which remained unchanged following treatment with empagliflozin (Figures 2J,K). Last, TazKD mice exhibited a decrease in both stroke volume and cardiac output when compared to their WT littermates, which was once again unaffected via treatment with empagliflozin (Figures 2L,M).

### Treatment with empagliflozin does not affect cardiac hypertrophy markers in TazKD mice

We assessed the relative mRNA expression of several markers of cardiac remodeling and hypertrophy. In general, TazKD mice exhibited increased or trends to increased myocardial mRNA expression of myosin heavy chain 7 (*Myh7*), skeletal muscle α-actin (*Acta1*), brain natriuretic peptide (*Nppb*), and atrial natriuretic peptide (*Nppa*) compared to their WT littermates (Figures 2N–Q). However, mRNA expression of these markers was unaffected via treatment with empagliflozin in TazKD mice, other than a trend to a mild reduction in *Nppb* expression (Figure 2P).

### Treatment with an oral KE increases circulating βOHB levels in TazKD mice but does not improve parameters of cardiac function and structure

**FIGURE 3:**
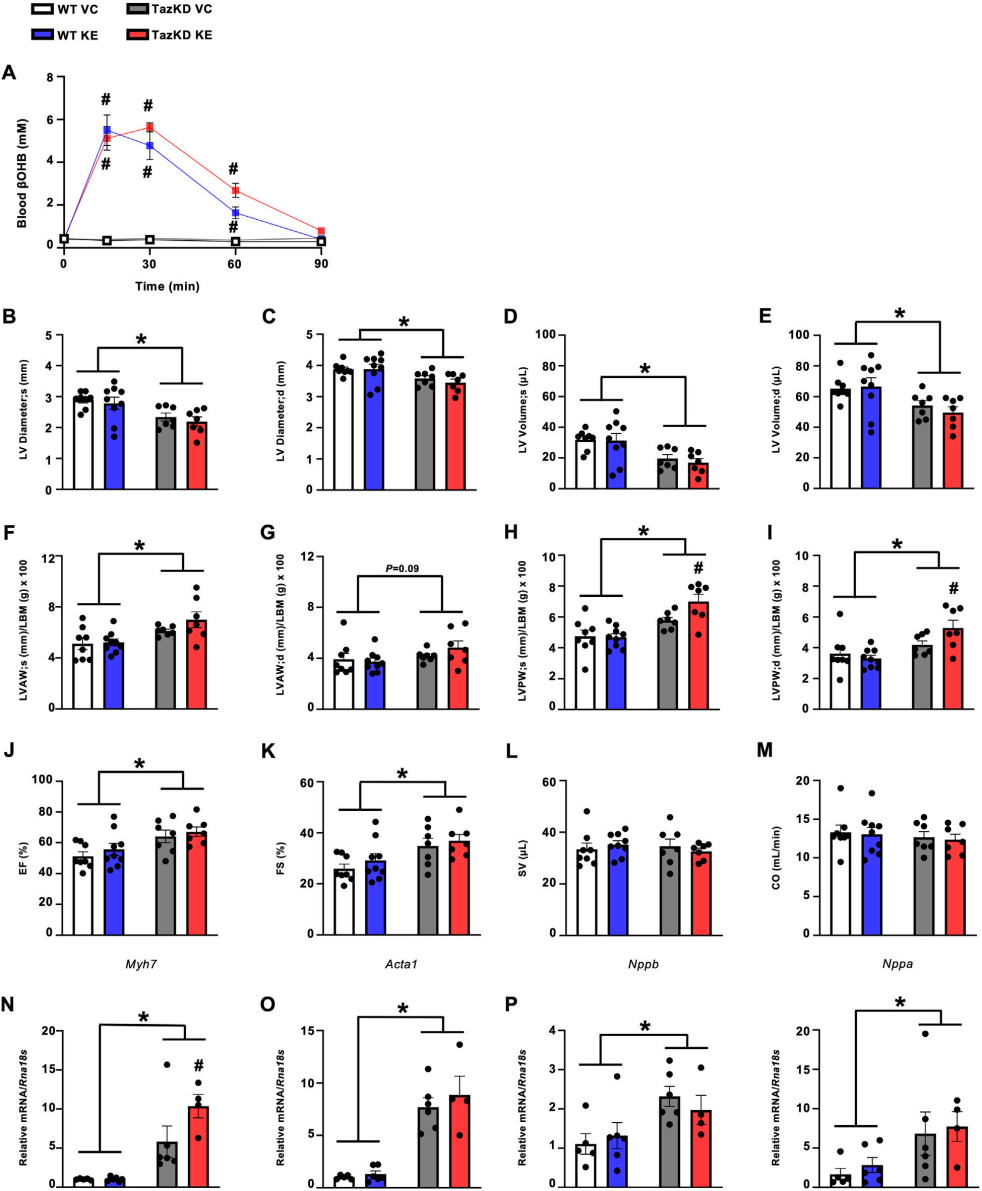
KE treatment increases circulating βOHB levels in fasted TazKD mice but does not alleviate the cardiac abnormalities in TazKD mice. **(A)** Circulating βOHB levels measured over 90 min following administration of an oral KE (n = 6–9). Ultrasound echocardiography was used to evaluate **(B,C)** LV diameter during systole and diastole, **(D,E)** LV volume during systole and diastole, **(F,G)** LVAW thickness during systole and diastole, **(H/I)** LVPW thickness during systole and diastole, **(J)** LVEF, **(K)** LVFS, **(L)** SV, and **(M)** CO in TazKD and WT mice treated with VC or an oral KE (n = 7–9). Myocardial mRNA expression of **(N)** *Myh7*, **(O)** *Acta1*, **(P)** *Nppb*, and **(Q)** *Nppa* relative to *Rna18s* (n = 4–6). All values are presented as mean ± SEM. *P < 0.05, significantly different versus WT mice. ^#^ *P* < 0.05, significantly different versus VC treated counterpart. *Acta1*, skeletal muscle α-actin; βOHB, β-hydroxybutyrate; CO, cardiac output; d, diastole; KE, ketone ester; LBM, lean body mass; LV, left ventricular; LVAW, left ventricular anterior wall; LVEF, left ventricular ejection fraction; LVFS, left ventricular fractional shortening; LVPW, left ventricular posterior wall; *Myh7*, myosin heavy chain 7; *Nppa*, atrial natriuretic peptide; *Nppb*, brain natriuretic peptide; *Rna18S*, 18S ribosomal RNA; s, systole; SV, stroke volume; VC, vehicle control; WT, Wild-type.

Because the empagliflozin mediated increase in circulating ketones was mild, we postulated that an approach to produce much larger increases in circulating ketones might be more likely to impact the cardiac abnormalities present in TazKD mice. As we have previously observed marked increases in circulating βOHB levels in mice treated with oral KEs [15, 22], we treated 7-week-old TazKD mice and their WT littermates with either an oral KE (1719 mg/kg) or VC via once daily oral gavage for 7-week. As expected, circulating βOHB levels were robustly increased to an equivalent extent over a 90-min duration in both TazKD and WT mice following administration of the KE (Figure 3A).

Once again opposing our hypothesis and recapitulating what we observed with empagliflozin treatment, KE administration for 7-week had no positive effects on the cardiac abnormalities present in TazKD mice. This includes no impact on LV diameter or LV volumes when compared to their VC treated TazKD counterparts (Figures 3B–E). However, the increased LV wall thickening that characterizes TazKD mice was mildly worsened via KE treatment, specifically in relation to the LV posterior wall (Figures 3F–I). With regards to systolic function, KE ester treatment of TazKD mice had no effect on LVEF, LVFS, stroke volume or cardiac output versus their VC treated counterparts (Figures 3J–M). It should be noted that this cohort of TazKD mice did not exhibit impairments in stroke volume or cardiac output, which others have reported [23, 24] and we have observed previously in these animals [20, 25], as well as in the empagliflozin cohort (Figures 2L,M). Reasons for these discrepancies in our 2 cohorts of TazKD mice are unclear, but could stem from the WT littermates in the KE arm of our study exhibiting more variability, particularly relating to their cardiac output.

### Treatment with an oral KE does not affect cardiac hypertrophy markers in TazKD mice

Similar to our previous observations, TazKD mice demonstrated increased mRNA expression of several markers of cardiac remodeling and hypertrophy. This included increased myocardial *Myh7*, *Acta1*, *Nppb*, and *Nppa* expression (Figures 3N–Q). Recapitulating what we observed following treatment with empagliflozin, KE administration did not impact the myocardial mRNA expression of these markers in TazKD mice, other than a mild exacerbation of *Myh7* expression (Figure 3N).

## Discussion

As a plethora of preclinical and clinical studies have demonstrated that cardiac substrate metabolism is altered in Barth syndrome [3], we aimed to determine whether increasing cardiac ketone metabolism may produce salutary actions against the cardiac phenotype present in TazKD mice. Recapitulating previous studies that have reported increased cardiac BDH1 expression and subsequent ketone metabolism in animals and humans with heart failure, we previously observed a robust increase in BDH1 protein expression in hearts from TazKD mice [20]. Accordingly, we posited that strategies to increase circulating ketone supply to the heart would improve outcomes in TazKD mice. In contrast to our hypothesis, both treatment with either the SGLT2 inhibitor, empagliflozin, or administration of an oral KE supplement increased circulating βOHB levels in TazKD mice, but failed to ameliorate the hypertrophic cardiomyopathy characterizing these animals.

Given the widely reported actions that SGLT2 inhibitors have salutary actions on cardiac function in type 2 diabetes and heart failure, which may be attributed to their ability to increase cardiac ketone metabolism [11], we were surprised by our findings in TazKD mice treated with empagliflozin. Voorrips et al. reported that empagliflozin treatment failed to improve LV function in cardiac-specific BDH1 knockout mice subjected to myocardial infarction via permanent left anterior descending coronary artery ligation, whereas beneficial effects were observed in their WT littermates [26]. Furthermore, administration of a KE alleviated LV dysfunction and cardiac hypertrophy in mice subjected to experimental heart failure secondary to transverse aortic constriction combined with an apical left anterior descending coronary artery ligation [22]. Empagliflozin was specifically chosen for this study given its clinical utility for the treatment of cardiovascular disease and its reported actions on ketone metabolism [11, 27]. Hence, the potential translation to humans with Barth syndrome could be meaningful had we observed positive outcomes in TazKD mice. While we are uncertain why empagliflozin was devoid of benefit in TazKD mice unlike the abovementioned studies in mice with experimental heart failure, it could stem from the fact that those studies mixed empagliflozin or the KE directly into the diet. In contrast, we administered empagliflozin once daily via oral gavage, and the overall increase in circulating ketone levels that we observed in TazKD mice (∼25% increase) was lower than what was reported by Voorrips and colleagues (∼100% increase) [26].

Because of the mild effect we observed on circulating ketones with empagliflozin, we decided to utilize a nutritional approach to increase ketones in TazKD mice, as we have previously observed robust increases in circulating βOHB levels in lean and obese mice administered an oral KE [15]. Despite recapitulating the robust increases in circulating ketones in TazKD mice via chronic treatment with an oral KE, we once again failed to observe any improvement in cardiac parameters of cardiac hypertrophy in TazKD mice. Nonetheless, it should also be noted that the increases in circulating ketones following KE administration were short-lived at ∼60–90 min. While that matches what has been observed in humans ingesting oral KEs [13], it remains possible that sustained increases in circulating ketones may be necessary to yield salutary actions on parameters of cardiac function. As such, adhering to a ketogenic dietary pattern may prove to be a better approach to achieve more sustained increases in circulating ketones in people living with BTHS, though this would come with its own set of limitations due to difficulties with long-term adherence to ketogenic diets. Another limitation of our study is that we performed our studies in relatively young TazKD mice, due to the fact that BTHS manifests in early life and is characterized by cardiomyopathy and/or heart failure throughout adolescence. However, it has been reported that the TazKD mouse model of BTHS exhibits a more late-onset cardiac phenotype at ∼7-month of age [16], and it will also be important to determine whether sustained increases in circulating ketones can prevent the progression of this late-onset cardiomyopathy.

We have previously reported that dichloroacetate treatment also failed to alleviate the hypertrophic cardiomyopathy in TazKD mice, even though it remained capable of increasing cardiac pyruvate dehydrogenase activity and glucose oxidation [25]. Taking our previous and current study into consideration, lends further support to the notion that unless the adverse CL remodeling and subsequent ETC defects are addressed, any therapeutic approach aimed at augmenting cardiac substrate metabolism is likely to be ineffective. It also remains possible that due to the TazKD mouse model exhibiting only a mild cardiac phenotype, we may be masking the full potential for metabolic therapies to improve the cardiac abnormalities that normally characterize BTHS. Another consideration is that while our treatments were chronic in nature as they were administered once daily for 7-week, the KE mediated increase in circulating βOHB levels only lasted for ∼90 min. Thus, it remains possible that formulations that lead to more sustained increases in ketones may have produced different results relating to cardiac outcomes.

Taken together, the present study demonstrates that pharmacological or dietary approaches to increase circulating ketones and subsequent ketone metabolism are ineffective against the hypertrophic cardiomyopathy that characterizes the TazKD mouse model of BTHS. Although the feasibility of testing SGLT2 inhibitors like empagliflozin, or using oral KEs in BTHS appeared promising due to the reported actions of these agents on cardiac function in humans with heart failure, such an approach cannot be recommended based on our observations. Further investigation is required to determine whether strategies to correct the ETC defects in TazKD mice are a necessity for any metabolic intervention to yield positive outcomes in treating BTHS-related cardiomyopathy.

## Data Availability

The raw data supporting the conclusion of this article will be made available by the authors upon reasonable request.

## Abbreviations

*Acta1*, skeletal muscle α-actin; BDH1, β-hydroxybutyrate dehydrogenase 1; BTHS, Barth syndrome; βOHB, β-hydroxybutyrate; CL, cardiolipin; CO, cardiac output; ETC, electron transport chain; KE, ketone ester; LBM, lean body mass; LV, left ventricular; LVAW, left ventricular anterior wall; LVEF, left ventricular ejection fraction; LVFS, left ventricular fractional shortening; LVPW, left ventricular posterior wall; *Myh7*, myosin heavy chain 7; *Nppa*, atrial natriuretic peptide; *Nppb*, brain natriuretic peptide; *Rna18s*, 18S ribosomal RNA; SGLT2, sodium-glucose cotransporter-2; SV, stroke volume; TazKD, *Tafazzin* knockdown; VC, vehicle control; WT, wild-type.
